# Limb Salvage Through Endovascular Repair of an Iatrogenic Superficial Femoral Artery Pseudoaneurysm Following Femoral Fracture Fixation

**DOI:** 10.7759/cureus.97378

**Published:** 2025-11-20

**Authors:** Zubair Farooq, Anees A Nengroo, Jasbir S Randhawa, Amit Sharma

**Affiliations:** 1 Cardiology, Randhawa Hospital and Trauma Center, Pathankot, IND; 2 Medicine, Randhawa Hospital and Trauma Center, Pathankot, IND; 3 Orthopedics, Randhawa Hospital and Trauma Center, Pathankot, IND

**Keywords:** case report, endovascular stent graft, limb salvage, post-traumatic vascular injury, superficial femoral artery pseudoaneurysm, threatened compartment syndrome

## Abstract

Vascular injury leading to pseudoaneurysm formation is a rare but potentially limb-threatening complication of lower limb trauma and postoperative recovery. We report a case of a 58-year-old man who developed progressive swelling of the right thigh one week after sustaining a road traffic accident and undergoing intramedullary nailing for a distal femoral shaft fracture. The swelling, initially presumed to represent a postoperative hematoma, progressed to cause severe tension and pain with early signs of threatened compartment syndrome. Upon transfer to our center, urgent surgical exploration for presumed hematoma evacuation revealed pulsatile bleeding suggestive of arterial injury. Computed tomography angiography, followed by invasive angiography performed the next morning, demonstrated a well-defined pseudoaneurysm arising from the superficial femoral artery, proximal to the knee joint.

The following day, definitive endovascular repair was performed via an antegrade right femoral approach. A 7 × 80 mm covered stent graft (Covera™ Plus Vascular Covered Stent; BD-Bard Peripheral Vascular Inc., Tempe, AZ, USA) was deployed across the arterial defect and post-dilated with a 7 × 40 mm Ultraverse™ PTA balloon (BD-Bard Peripheral Vascular Inc., Tempe, AZ, USA), achieving complete exclusion of the pseudoaneurysm while maintaining normal distal perfusion. The procedure was completed under local anesthesia without complications. Postoperatively, the patient demonstrated rapid clinical improvement with marked reduction in thigh swelling, pain, and compartmental tension. Follow-up imaging confirmed stent patency and complete exclusion of the pseudoaneurysm. At three months, he had full functional recovery and was able to bear weight independently.

This case underscores the importance of recognising vascular injury as a cause of progressive postoperative limb swelling, particularly when accompanied by increasing pain and tension suggestive of impending compartment syndrome. Early vascular imaging, including computed tomography angiography followed by confirmatory invasive angiography when necessary, is crucial for accurate diagnosis. Endovascular covered stent grafting provides a safe, minimally invasive, and limb-saving alternative to open surgery, allowing rapid hemostasis, preservation of arterial flow, and excellent long-term outcomes.

## Introduction

Iatrogenic vascular injuries after orthopedic procedures are uncommon, but when they occur, they can lead to limb-threatening complications. Pseudoaneurysm formation in the femoral vessels following intramedullary femoral nailing is exceedingly rare, with only a handful of cases reported in the literature [1]. A pseudoaneurysm (false aneurysm) is a contained arterial rupture where blood leaks out of the vessel and is confined by surrounding tissue, lacking the full tri-layered arterial wall. This condition may present insidiously, often masquerading as a simple postoperative hematoma or deep vein thrombosis (DVT), making diagnosis challenging [2]. In the context of femoral fracture fixation, the superficial femoral artery (SFA), profunda femoris artery, or their branches (such as the lateral circumflex femoral artery) may be injured by fracture fragments, drilling of locking screws, malpositioned guidewires, or retractor pressure [3].

Vascular injury during femoral fracture fixation is uncommon, with only isolated reports describing pseudoaneurysm formation involving the superficial or profunda femoris arteries. Although rare, such injuries can be limb-threatening if unrecognized. Historically, open surgical repair was the standard management for such vascular injuries. However, advances in endovascular techniques (coil or glue embolization, covered stent grafting) now offer less invasive alternatives with favorable outcomes and lower morbidity [1]. We present a case of a post-traumatic SFA branch pseudoaneurysm developing after distal femoral intramedullary nailing, emphasizing the diagnostic dilemmas, the critical role of early CTA imaging, and the successful endovascular management. We also review prior cases of pseudoaneurysm related to orthopedic procedures and compare endovascular repair versus open surgical treatment in terms of outcomes and technical considerations.

## Case presentation

A 58-year-old South Asian male presented following a high-speed road traffic accident with an isolated fracture involving the distal one-third of the right femur. He underwent urgent intramedullary nailing of the femur at a peripheral hospital on the day of injury (Day 0). The procedure was reported as uneventful, with distal locking screws applied. The postoperative period was initially stable, and the limb remained neurovascularly intact.

Over the subsequent week, the patient developed progressively increasing diffuse swelling of the right thigh associated with severe pain and a sensation of tightness. The swelling, initially presumed to represent postoperative hematoma or edema, continued to worsen and was accompanied by increasing tension and discomfort suggestive of early compartmental compromise. There was no external bleeding or wound discharge, and distal pulses (dorsalis pedis and posterior tibial) remained palpable.

By postoperative Day 7, the progressive swelling and pain prompted transfer to our tertiary-care center for further evaluation of a suspected expanding hematoma or threatened compartment syndrome. On admission (Day 8), the patient was hemodynamically stable but in significant pain. The right thigh appeared markedly swollen, tense, and shiny, with diffuse fullness involving its entire circumference. There was no localized pulsatility or bruit on auscultation, but the thigh was firm and tender, raising concern for an underlying arterial injury and evolving compartment syndrome. Peripheral pulses were present, and the limb remained warm, though under marked tension.

Given the clinical picture, urgent surgical exploration was planned for presumed hematoma evacuation. Intraoperatively, after making a lateral thigh incision and entering the intramuscular plane, a large volume of organized clot was encountered. During evacuation, brisk pulsatile arterial bleeding was suddenly noted from deep within the thigh muscles, indicating an arterial source. The procedure was immediately aborted, hemostasis was achieved by tight packing, and the vascular surgery and radiology teams were urgently consulted.

**Figure 1:**
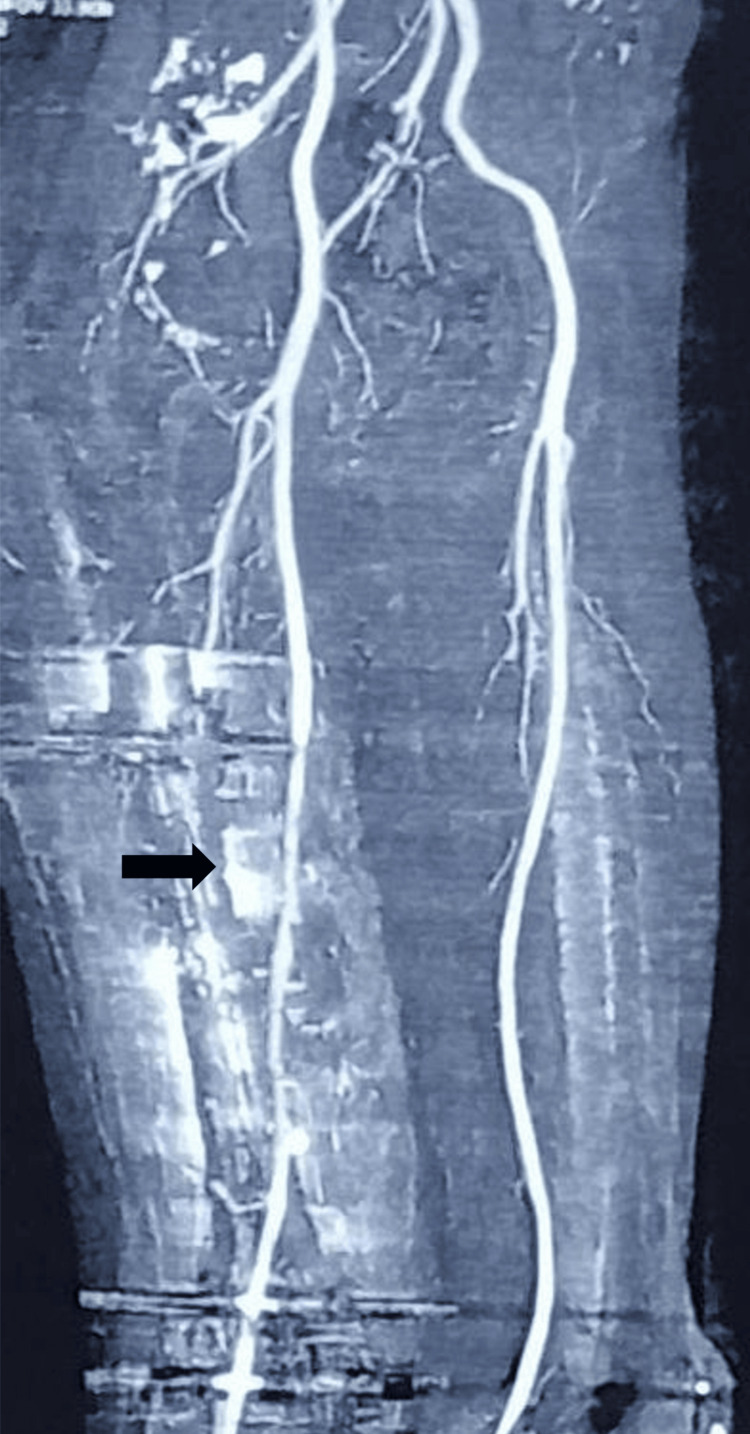
CT angiography of the thigh showing a suspected pseudoaneurysm of the superficial femoral artery (black arrow).

A contrast-enhanced CT (Computed Tomography) angiography performed later that evening suggested the presence of a pseudoaneurysm arising from the superficial femoral artery (SFA) in the mid-to-distal thigh, though the exact site of arterial breach could not be confidently localized (Figure 1).

**Figure 2:**
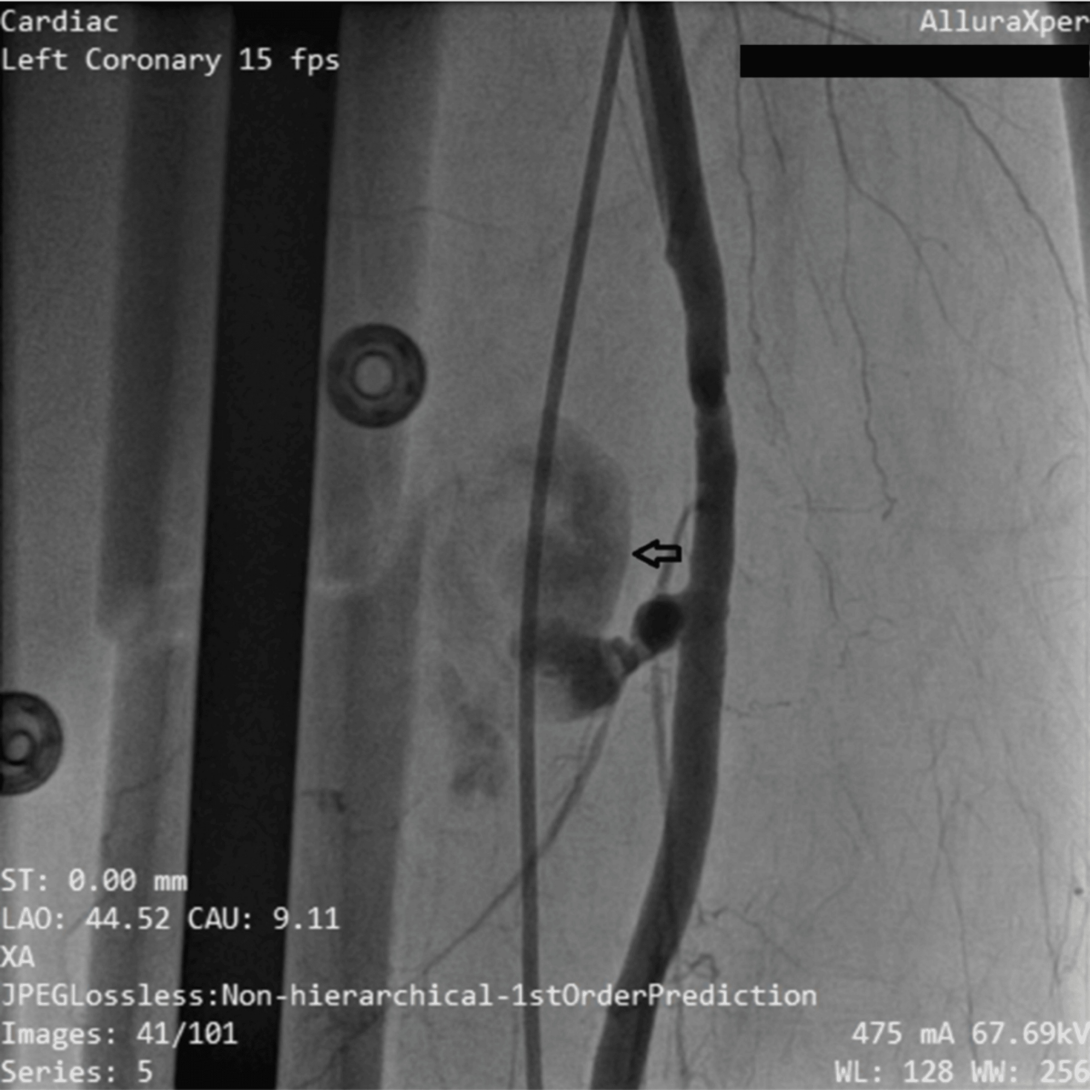
Cine angiographic view via contralateral femoral access demonstrating right superficial femoral artery pseudoaneurysm (black arrow).

The following morning (Day 9), invasive angiography was performed via contralateral access (left common femoral artery) showed a large pseudoaneurysm arising directly from the superficial femoral artery with active contrast extravasation into the pseudoaneurysm cavity, confirming the diagnosis (Figure 2).

**Figure 3:**
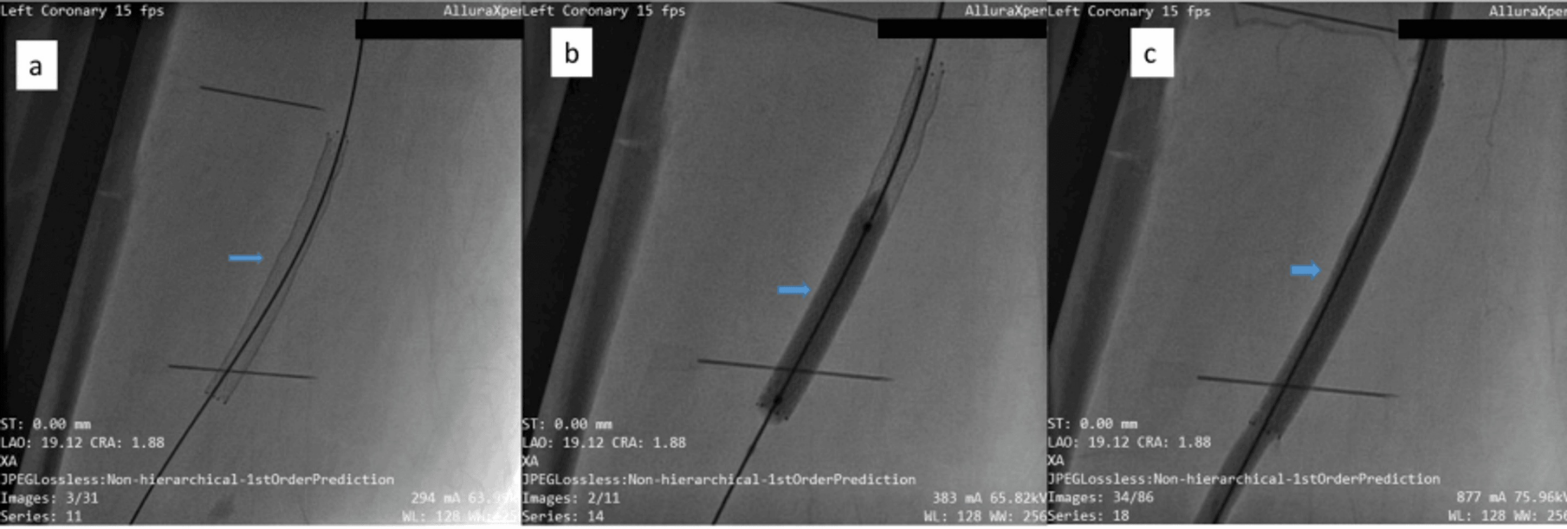
Sequential angiographic views demonstrating endovascular repair and successful exclusion of the pseudoaneurysm (blue arrows) (a) Cine angiogram showing deployment of the covered graft across the site of the pseudoaneurysm. (b) Cine frame obtained during post-dilatation of the deployed graft to ensure complete apposition to the vessel wall. (c) Final angiogram demonstrating complete sealing of the rupture with no contrast extravasation, confirming successful exclusion of the pseudoaneurysm.

On the same day (Day 9), definitive endovascular repair was performed under local anesthesia. An 8-French vascular sheath (Radifocus® Introducer II, Terumo Corporation, Tokyo, Japan) was introduced, and the lesion was crossed using a 0.035-inch hydrophilic guidewire (Glidewire®, Terumo Corporation, Tokyo, Japan) and a Judkins Right (JR 3.5) diagnostic catheter (Cordis, Miami Lakes, FL, USA). A 7 × 80 mm covered stent graft (Covera™ Plus Vascular Covered Stent; BD-Bard Peripheral Vascular Inc., Tempe, AZ, USA) was advanced over the Terumo wire and deployed across the arterial defect (Figure 3a). Post-dilatation was carried out using a 7 × 40 mm Ultraverse™ PTA balloon (BD-Bard Peripheral Vascular Inc., Tempe, AZ, USA) to ensure adequate apposition and sealing of the lesion (Figure 3b). Completion angiography demonstrated complete exclusion of the pseudoaneurysm with preservation of distal perfusion and no residual leak (Figure 3c).

The previously seen contrast extravasation had ceased, and antegrade flow through the SFA into the popliteal artery was maintained. The entire endovascular procedure was completed in approximately 60 minutes with an estimated blood loss of less than 50 mL.

Systemic heparin (10,000 IU) was administered during the procedure. The femoral puncture site was sealed with manual compression, and hemostasis was achieved without complication. The patient was monitored overnight in a high-dependency unit. Post-procedure, heparin was discontinued, and dual antiplatelet therapy with aspirin and clopidogrel was initiated after appropriate loading doses. A high-intensity statin was also started as part of the vascular protection protocol to maintain stent patency and optimize endothelial recovery.

Following the intervention, there was a rapid reduction in thigh swelling and resolution of compartmental tension. The limb remained warm and well-perfused, and distal pulses were palpable. The postoperative course was smooth, with no evidence of distal ischemia or access-site complications. At three months of follow-up, the patient had regained full weight-bearing ability, with no recurrence of swelling, normal limb perfusion, and a patent stent on duplex ultrasonography. The timeline of events is shown in Table 1.

**Table 1:** Timeline of clinical events and interventions in a case of post-traumatic superficial femoral artery pseudoaneurysm.

Timeline	Clinical events and interventions
Day 0	High-speed road traffic accident resulting in an isolated fracture of the distal one-third of the right femur. Urgent intramedullary nailing was performed at a peripheral hospital. Immediate postoperative period uneventful; limb neurovascularly intact.
Days 1 – 6	Progressive diffuse swelling, pain, and tightness of the right thigh gradually increasing; initially attributed to postoperative hematoma or edema.
Day 7	Worsening thigh swelling and pain with increasing tension and discomfort suggestive of threatened compartment syndrome. Planned exploration for a suspected expanding hematoma.
Day 8	Lateral thigh exploration was performed for presumed hematoma evacuation. Large organized clot encountered, followed by brisk arterial bleeding from deep muscle planes. Procedure aborted; hemostasis achieved by tight packing. CT angiography suggested a pseudoaneurysm of the superficial femoral artery (SFA) in the mid-to-distal thigh.
Day 9 (Morning)	Invasive angiography confirmed a 25.8 × 23 mm pseudoaneurysm arising from the SFA with active contrast extravasation.
Day 9 (Later)	Definitive endovascular repair under local anesthesia. Procedure performed via antegrade right common femoral arterial puncture under fluoroscopic guidance using an 8-French sheath, a 0.035-inch Terumo hydrophilic guidewire, and a Judkins Right (JR 3.5) catheter. A 7 × 80 mm Covera Plus covered stent graft was deployed across the arterial defect and post-dilated with a 7 × 40 mm Ultraverse balloon. Completion angiogram showed complete exclusion of the pseudoaneurysm and preserved distal flow.
Day 9 (Post-procedure)	Systemic heparin 10,000 IU administered intra-procedure. Puncture site sealed with manual compression. Monitored overnight in a high-dependency unit. Dual antiplatelet therapy (aspirin + clopidogrel after loading doses) and a high-intensity statin were initiated.
Day 10 onward	Rapid improvement with reduction in swelling and resolution of compartmental tension. Limb warm and well-perfused; distal pulses palpable. No ischemic or access-site complications.
3-Month follow-up	Full weight-bearing achieved without pain. Duplex ultrasound confirmed patent stent and normal limb perfusion. Excellent functional recovery.

## Discussion

Pseudoaneurysm formation following orthopedic trauma or surgery results from a partial arterial wall injury that leads to blood dissection into surrounding tissues, forming a contained hematoma. Unlike a true aneurysm (which involves all vessel wall layers), a pseudoaneurysm is bounded only by adventitia or surrounding tissue [4]. In femoral fractures, a sharp bone fragment or surgical instrument (e.g. drill bit or screw) can lacerate or weaken an arterial wall, while the intact portion of the vessel prevents full transection. Blood leaks through the partial tear and organizes into a fibrous capsule, creating a pulsatile cavity connected to the artery. Over time, the ongoing arterial flow maintains the pseudoaneurysm, which may enlarge under systemic pressure [4].

In the superficial femoral artery (SFA), which traverses the distal thigh (adductor canal), proximity to distal femoral fractures means bone spike injuries or retraction during surgery can damage the vessel. Atherosclerotic arterial walls (common in older patients) are especially fragile; even minor trauma or stretching during nailing can precipitate intimal damage and pseudoaneurysm formation. Notably, the absence of an initial free hemorrhage (due to tamponade by surrounding tissue) allows pseudoaneurysms to form insidiously. In summary, the pathophysiology involves an incomplete arterial rupture leading to a contained hematoma that fails to resorb and instead pulsates with arterial flow, gradually expanding if not recognized [2].

Femoral artery pseudoaneurysms typically present in a delayed fashion, ranging from days to even years post-injury [5]. In the orthopedic setting, patients often develop progressive thigh swelling, fullness, or pain at the fracture or surgical site after an initially uneventful recovery. Pulses distal to the injury are usually preserved (since flow continues through the pseudoaneurysm or via collaterals), so classic “hard signs” of acute vascular injury (absent pulses, ischemia) are often absent. Instead, patients may have only “soft signs” such as a vague mass, chronic hematoma, or minor bruising. This was reflected in the present case; the patient’s increasing thigh girth and pain were initially attributed to a postoperative hematoma, delaying diagnosis. Such diagnostic delay is common; pseudoaneurysm symptoms overlap with more benign post-operative issues like hematoma, seroma or deep vein thrombosis (DVT). In several reported cases, swelling was mistaken for infection or muscle edema, as a contained pseudoaneurysm can cause inflammatory changes (warmth, tenderness) mimicking abscess or hematoma organization. A high index of suspicion is therefore crucial [6].

The unexpected involvement of the SFA in a distal femur fracture highlights how even well-understood surgical sites can harbor surprising vascular risks. Clinically, the presence of a pulsatile mass, a palpable thrill, or an audible bruit on auscultation should raise concern for pseudoaneurysm despite normal distal perfusion. In the absence of these findings, imaging is key. Duplex Doppler ultrasound can readily identify a pseudoaneurysmal sac with turbulent flow, while contrast-enhanced CT angiography provides precise localization and size of the lesion. In the present case, once an arterial injury was considered, a CT angiogram promptly confirmed a mid-thigh SFA pseudoaneurysm that explained the patient’s symptoms. Vascular complications from orthopedic procedures are fortunately rare.

Over the last 10-15 years, multiple case reports and small series have documented femoral artery pseudoaneurysms following intramedullary nailing or plate fixation of femoral fractures. For example, Piolanti et al. (2017) reported a profunda femoris pseudoaneurysm after cephalomedullary nailing of a pertrochanteric fracture [7], and Bowden et al. (2020) described a large DFA pseudoaneurysm one month after hip screw fixation [8]. In contrast, pseudoaneurysms of the superficial femoral artery are less frequent, but have been documented especially with distal femur injuries. Lo et al. (2011) reported an SFA pseudoaneurysm presenting six weeks after distal femoral shaft fracture fixation [9]. Their patient developed a posteromedial thigh mass that was ultimately found to be an SFA pseudoaneurysm, treated by endovascular stent grafting followed by open hematoma evacuation. Hirota et al. (2014) likewise described an SFA pseudoaneurysm after retrograde intramedullary nailing of a supracondylar femur fracture [10]. In that case, the pseudoaneurysm was managed with surgical intervention after delayed recognition. 

This case serves as a reminder that in orthopedic trauma, subtle postoperative signs may conceal serious vascular complications, reinforcing the value of timely imaging and multidisciplinary collaboration. Our present case shares similarities with these reports, all involved distal femoral fractures with insidious SFA pseudoaneurysm formation weeks to months later. Notably, direct SFA injury in distal femur fracture is unusual; as highlighted by a recent series, femoral pseudoaneurysm in distal-third fractures “is most commonly seen in the profunda femoris artery… our case had the SFA involved” [4]. This underscores the rarity of the complication we encountered. Nonetheless, clinicians should be aware that even the SFA can be jeopardized by distal femoral screws, plates, nail insertion, or bony fragments.

Other published cases have broadened the spectrum of arterial injuries in femur fractures: for instance, Vande Voorde et al. (2018) reported a delayed profunda femoris pseudoaneurysm after femoral nailing [11], and Ali et al. (2014) documented an SFA pseudoaneurysm caused by a condylar buttress plate screw [12]. The common lesson from these cases is the need for vigilance long after the initial orthopedic procedure. All authors stress maintaining a high suspicion for vascular injury when patients present with atypical pain or swelling after fracture fixation. In summary, while femoral artery pseudoaneurysm is a rare complication of musculoskeletal trauma care, it is well-documented. Our case contributes to this body of literature by highlighting an SFA pseudoaneurysm after distal femur nailing, an infrequent location, and reinforces trends noted in prior cases (delayed presentation, masquerading symptoms, and successful outcomes with appropriate intervention). Management of femoral pseudoaneurysms must be individualized, taking into account the pseudoaneurysm’s size, location, and the patient’s condition. Both endovascular techniques and open surgical repair are employed, often complementing each other. In all cases, prompt control of bleeding and prevention of rupture are primary goals.

Key treatment modalities

Endovascular Stent Grafting

Covered stent grafts can be deployed across the arterial defect to exclude the pseudoaneurysm while preserving flow through the parent artery [9]. This approach is especially valuable for injuries to the SFA or other major arteries where maintaining limb perfusion is critical. The major advantages are minimally invasive deployment (via angiography) and avoidance of a surgical incision in a potentially scarred or infected field. Stent grafting has been successfully used in several reports of femoral pseudoaneurysm, including the present case, where a covered stent achieved complete exclusion of the SFA pseudoaneurysm with preservation of distal circulation. By sparing the vessel, stenting reduces the risk of limb ischemia. However, drawbacks include the need for prolonged dual antiplatelet therapy to maintain stent patency and the risk of stent thrombosis or occlusion over time. There is also a concern for infection of the graft, particularly if it lies near an orthopedic hardware site or in a hematoma that could be contaminated. In our case, careful sterile technique and the absence of overt infection allowed a stent graft to be placed with an excellent outcome (patent at 3-month follow-up). Stent grafting is generally indicated when the artery is large and vital (e.g. SFA or common femoral) and when local sepsis is not present.

Endovascular Embolization

For pseudoaneurysms arising from smaller branches or in situations where sacrificing the injured artery is acceptable, embolization is a favored minimally invasive option. Embolization can be achieved with coils, plugs, or even liquid agents (glue/onyx), delivered via catheter to occlude the pseudoaneurysm sac or its feeding artery. The classic example is coil embolization of a profunda femoris artery pseudoaneurysm; since the superficial femoral artery remains intact, the profunda can often be occluded without major limb ischemia. Several case studies document successful coil embolization of profunda pseudoaneurysms after hip fracture fixation. In the present case, embolization was considered; however, given the pseudoaneurysm involved the SFA (the primary arterial supply to the lower leg), permanently occluding the vessel was undesirable. Instead, a covered stent preserved SFA continuity. Embolization is best reserved for branch vessels or situations where collateral circulation can compensate. Its advantages include high success rate in stopping hemorrhage and avoiding open surgery. The downsides are the intentional loss of the target artery’s flow and the possibility of collateral damage or migration of embolic material. In a recent Cureus case report of a lateral femoral circumflex artery pseudoaneurysm after femur nailing, initial ultrasound-guided compression failed and the pseudoaneurysm was ultimately treated with embolization (using glue) with good effect [1]. This illustrates that feasible, embolization offers a safe and effective cure for iatrogenic pseudoaneurysms with minimal morbidity.

Open Surgical Repair

Traditionally, open surgery was the definitive treatment for traumatic pseudoaneurysms, and it remains indispensable in certain scenarios. Open repair involves surgical exposure of the artery, ligation or clamping of inflow, excision of the pseudoaneurysm sac, and reconstruction of the vessel as needed. Techniques include primary arterial repair (lateral wall suturing), patch angioplasty, interposition grafting with vein, or simply ligation of the vessel if perfusion can be maintained by collaterals [10]. The chief advantage of open surgery is direct control of the hemorrhage and the ability to evacuate the hematoma, relieving mass effect on nerves or veins. The disadvantages, however, include surgical morbidity (larger incision, blood loss) and challenges in accessing deep vessels amid scar tissue or hardware. In reported cases, open repair has yielded good outcomes, especially when performed promptly [5].

The present case exemplifies several key themes noted in prior reports while also highlighting unique considerations. First, it featured direct SFA involvement, whereas many femoral pseudoaneurysms after fracture fixation involve the profunda femoris. This direct injury to the SFA is relatively uncommon [4], making our case an important addition to the limited reports of SFA pseudoaneurysm in orthopedic trauma. The mechanism was likely an iatrogenic arterial wall injury during intramedullary nailing of the distal femur - potentially from a distal locking screw or reamer encroaching on the SFA’s course. A similar mechanism was proposed by Grimaldi et al. (2009), who described an SFA injury from a distal locking screw in an intertrochanteric nailing [13].

Secondly, our case underscores the diagnostic challenge: the pseudoaneurysm’s early manifestations were misinterpreted as a postoperative hematoma for about one week. Such delays are well-documented; most cases in the literature were not recognized until the patient developed significant swelling or hemorrhage days to weeks later. In our case, the tipping point was the development of extreme thigh tightness and pain by Day 7 post-op, suggestive of compartment syndrome (due to the enlarging pseudoaneurysm compressing compartments). This prompted surgical exploration. Interestingly, this contrasts with some reports where early imaging (ultrasound or CT) was obtained in time to diagnose the pseudoaneurysm noninvasively. The fact that our patient went to the operating room with a presumed hematoma and that only during surgery was the arterial injury discovered, highlights the potential for pseudoaneurysms to mimic benign postoperative collections. Once arterial bleeding was seen intra-operatively (a brisk spurting from the depth of the clot), appropriate steps were taken, including packing for hemostasis and urgent vascular imaging. This sequence of emergency hematoma evacuation, revealing a pseudoaneurysm, has been described in other cases where a pseudoaneurysm was initially missed. Dhal et al. (2001) reported a series of delayed vascular injuries in orthopedic practice, noting that surgical evacuation of what was thought to be an abscess/hematoma sometimes unmasked an arterial bleeder [14]. The lesson is clear: orthopedic surgeons should consider a pseudoaneurysm in any post-fixation hematoma that is unusually large, pulsatile, or unresolving. In retrospect, subtle signs (such as a faint bruit or an atypical location of the hematoma) might alert clinicians before such a dramatic reveal.

Finally, the management and outcome of this case deserve comment. We achieved successful thrombosis and exclusion of the pseudoaneurysm using a covered stent graft in the SFA, thereby preserving arterial continuity. This minimally invasive solution aligns with the growing trend favoring endovascular repair for such complications. The patient’s rapid recovery, resolution of swelling and full limb perfusion along with maintained stent patency, demonstrate the effectiveness of endovascular management for pseudoaneurysms, even in emergency settings.

## Conclusions

Pseudoaneurysm of the superficial femoral artery is a rare but serious complication following femoral fracture fixation that can mimic postoperative hematoma. High clinical suspicion and early vascular imaging are essential for timely diagnosis. Endovascular covered stent grafting provides an effective, minimally invasive option that controls bleeding, preserves arterial patency, and facilitates rapid recovery. Prompt recognition and multidisciplinary coordination remain vital for limb salvage and favorable long-term outcomes.
